# Quantification and Localization of *Watermelon Chlorotic Stunt Virus* and *Tomato Yellow Leaf Curl Virus* (*Geminiviridae*) in Populations of *Bemisia tabaci* (*Hemiptera*, *Aleyrodidae*) with Differential Virus Transmission Characteristics

**DOI:** 10.1371/journal.pone.0111968

**Published:** 2014-11-03

**Authors:** Mario Kollenberg, Stephan Winter, Monika Götz

**Affiliations:** Plant Virus Department, Leibniz Institute DSMZ-German Collection of Microorganisms and Cell Cultures, Braunschweig, Germany; Zhejiang University, China

## Abstract

*Bemisia tabaci* (Gennadius) is one of the economically most damaging insects to crops in tropical and subtropical regions. Severe damage is caused by feeding and more seriously by transmitting viruses. Those of the genus begomovirus (*Geminiviridae*) cause the most significant crop diseases and are transmitted by *B. tabaci* in a persistent circulative mode, a process which is largely unknown. To analyze the translocation and to identify critical determinants for transmission, two populations of *B. tabaci* MEAM1 were compared for transmitting *Watermelon chlorotic stunt virus* (WmCSV) and *Tomato yellow leaf curl virus* (TYLCV). Insect populations were chosen because of their high and respectively low virus transmission efficiency to compare uptake and translocation of virus through insects. Both populations harbored *Rickettsia, Hamiltonella* and *Wolbachia* in comparable ratios indicating that endosymbionts might not contribute to the different transmission rates. Quantification by qPCR revealed that WmCSV uptake and virus concentrations in midguts and primary salivary glands were generally higher than TYLCV due to higher virus contents of the source plants. Both viruses accumulated higher in insects from the efficiently compared to the poorly transmitting population. In the latter, virus translocation into the hemolymph was delayed and virus passage was impeded with limited numbers of viruses translocated. FISH analysis confirmed these results with similar virus distribution found in excised organs of both populations. No virus accumulation was found in the midgut lumen of the poor transmitter because of a restrained virus translocation. Results suggest that the poorly transmitting population comprised insects that lacked transmission competence. Those were selected to develop a population that lacks virus transmission. Investigations with insects lacking transmission showed that virus concentrations in midguts were reduced and only negligible virus amounts were found at the primary salivary glands indicating for a missing or modified receptor responsible for virus attachment or translocation.

## Introduction

The whitefly *Bemisia tabaci* (Gennadius) (Hemiptera: Aleyrodidae) is an agricultural pest in tropical and subtropical regions. It is a polyphagous phloem-feeder that causes damage in many crops due to direct feeding and vectoring of numerous plant viruses [1], [2]. The majority of the transmitted viruses are from the genus *Begomovirus* (family *Geminiviridae* [3], [4]; some of which cause serious diseases with high economic impact [5]. *B. tabaci* is a complex of at least 24 cryptic species, and considerable biological and genetic variation exists among natural populations within each species [6]–[8]. Most prominently, species vary considerably in their ability to feed on different hosts and also in the efficiency to transmit viruses [1], [9]. Begomoviruses have circular single-stranded DNA genomes encapsidated in geminate particles [10]. *Tomato yellow leaf curl virus* (TYLCV) has a monopartite genome [29] while that of *Watermelon chlorotic stunt virus* (WmCSV) is bipartite with a DNA-A and a DNA-B genomic component [11]. Both cause serious crop diseases: WmCSV threatens the production of cucurbit crops like watermelon (*Citrullus lanatus*), melons (*Cucumis melo*), and pumpkin (*Cucurbita moschata*) in North Africa, Yemen, Israel, Jordan and Iran [12]. Disease symptoms include vein yellowing, chlorotic mottling and stunting and severe curling of leaves with drastic reduction of yield [13], [14]. TYLCV is the major constraint to tomato (*Solanum lycopersicum*) production worldwide and losses in fruit yield up to 100% are reported [15] especially when young plants become infected. Symptoms on tomato consist of a more or less pronounced upward curling of leaflets and leaf yellowing which is most prominent on young leaves in the apical parts of the plants.

Begomoviruses are exclusively vectored by *B. tabaci* in a circulative persistent manner [15], [16]. Virus particles are acquired with the phloem sap of infected host plants during feeding and pass through the esophagus to reach the filter chamber of the insect [17]. Filter chamber and anterior region of the midgut are the sites of virus translocation into the hemolymph where virions bind to GroEL, a chaperone produced by bacterial endosymbionts, that protects them from degradation by the harsh conditions in the hemolymph [18], [19]. Virions circulate within the hemolymph to reach and enter into the primary salivary glands (PSG) to finally be excreted into the salivary duct and injected to infect new host plants [20]. This translocation process is described in detail by [21].

During the translocation from the midgut into the hemocoel and from the hemolymph into the PSG and salivary duct the viruses have to overcome several barriers: the outer and inner membranes of the gut and the membranes of the PSG. Because virus/vector interactions are very specific, recognition of virus (coat protein) and whitefly receptors is prerequisite for virion passage through the insect. Thus the ability for virus transmission is supposed to be an inheritable trait.

Differences in virus transmission efficiencies were also found for other insects and virus/vector combinations. Rosa and Kennedy [22] showed that the ability of *Thrips tabaci* to transmit *Tomato spotted wilt virus* differs significantly between populations and that this is inherited as a recessive trait. In populations of the aphid *Schizaphis graminum* different transmission efficiencies of *Cereal yellow dwarf virus* also were attributed to inheritable traits regulated by several unlinked genes [23].

In the present study we compared two whitefly populations, one efficiently transmitting begomoviruses and the other, with low capability of begomovirus transmission, to compare uptake and translocation of WmCSV and TYLCV. Quantitative PCR (qPCR) and *in situ* hybridization were used to quantify and localize virus in insects, in hemolymph and in excised midguts and PSG to identify and resolve sites critical to virus translocation in vector insects.

## Material and Methods

### Insects and rearing conditions

Two laboratory populations of *B. tabaci* MEAM1 [24], [25] initially collected from infested squash plants in Gezira (Sudan) in 2002 were maintained in insect-proof cages on cotton seedlings (*Gossypium hirsutum*) at 26°C and a photoperiod of 14 h. The whitefly populations were collected during a survey in open fields to assess the molecular and biological diversity of the insects which are important agricultural pests and virus vectors. A permit for collection was not required whereas a permit was issued from the German authorities to work with these insects which are regulated under EU Annex I/A1 (non-European populations).

Viruses were maintained on watermelon (*Citrullus lanatus* cv. Sugarbaby) serving as host for WmCSV and on tomato (*Solanum lycopersicum* cv. Linda) infected with TYLCV.

Both insect populations were typified as cryptic species MEAM1 on the basis of sequencing the ribosomal DNA internal transcribed spacers ITS1 and ITS 2 [26] and a fragment of the mitochondrial cytochrome oxidase subunit I [27].

Transmission rates were determined by transferring single viruliferous whiteflies (acquisition access period, AAP 3 d) to host plants (inoculation access period, IAP 7 d) and assessment of symptoms 28 days after inoculation. For TYLCV, high infection rates (96.6%) were determined for population *B. tabaci* 63 and poor efficiencies were recorded for population *B. tabaci* 95 (15.0%). Infection rates for WmCSV were lower compared to TYLCV with 32.4% when inoculated with *B. tabaci* 63 and 9.1% when inoculated with groups of 10 *B. tabaci* 95 individuals per watermelon plant.

All experiments in this study were performed with synchronized whitefly populations as virus acquisition and transmission efficiency decrease with the insect age [28]. Viruliferous whiteflies were discharged after a five day AAP by rearing them on cotton plants for two days except otherwise stated.

### Viruses, and agroinoculation assays

Infectious clones of WmCSV corresponding to dimers of DNA-A and DNA-B genomic components in pBin19 [11], kindly provided by Dr. Bruno Gronenborn (CNRS, France) and an infectious clone of TYLCV, comprising a dimerized virus genome in pcGN1547 [29] kindly supplied by Dr. Henryk Czosnek (The Hebrew University of Jerusalem, Israel) were used to establish virus infections. Agroinoculation using *Agrobacterium tumefaciens* strain LBA 4404 harboring cloned genomes of the respective viruses was by needle injection of bacterial suspensions essentially as described [30] into stems of tomato and watermelon plants at the two leaf and three leaf stage, respectively. Treated plants were maintained under greenhouse conditions at 26°C and a 14 h light period under strict containment conditions. Symptoms developed about two weeks after inoculation.

### DNA extraction from *B. tabaci* and host plants

Whitefly individuals were collected with an aspirator and stored at −80°C until DNA extraction. DNA from groups of 50 to 100 insects was extracted following a CTAB protocol [31] including RNase treatment. Total DNA extraction from whitefly individuals was performed following the protocol of Frohlich et al. [27]. For virus assays in plants, DNA was extracted using the DNeasy Plant Mini Kit (QIAGEN, Germany) following the manufacturer's instructions. DNA concentrations were measured photometrically (NanoDrop Spectrophotometer ND-1000, Peqlab Biotechnologie GmbH, Germany). Midguts and salivary glands were excised from single insects and groups of 10 PSG were subjected immediately after excision to qPCR, immersing the organs in 10 µl double distilled water and boiling at 95°C for 6 min for cell disruption. Hemolymph was drawn from insects using a glass capillary and liquid from 5 females was subjected to qPCR.

### Identification of secondary bacterial endosymbionts

DNA of twenty single male and female individuals per population was extracted following the protocol of Frohlich et al. [27] and subjected to PCR analysis according to Chiel et al. [32]. PCR products were ligated into pDrive (QIAGEN, Germany) and transferred into *Escherichia coli* DH5α. Sequencing was done by Eurofins MWG Operon (Germany) and analyzed using Vector NTI Advance software 11.0 (Invitrogen, Germany).

### Assessment of ingestion rates

Virus uptake was assessed in three independent experiments. Three to five days old whiteflies were transferred to WmCSV infected plants after six hours of starvation. Insects were collected 0, 1, 2, 16 h and 5 days after transfer to measure virus concentration. For qPCR, DNA was extracted from groups of 100 insects and virus concentration was related to one insect after qPCR.

### Quantification of WmCSV and TYLCV in whiteflies

Three to five days old whiteflies were discharged for two days after a 5 day AAP on virus infected source plants. Whiteflies maintained on healthy plants served as controls. Virus concentrations quantified by qPCR were determined for whole whiteflies (100 individuals per sample) and excised midguts as well as primary salivary glands (10 PSG per sample). To study virus translocation into the hemolymph three to five days old whiteflies were starved for 2 h and subsequently transferred to virus infected host plants. Hemolymph was collected 0, 2, 4, 6, 8, 24, 30 h and 6 days after transfer. Hemolymph liquid of 5 females was pooled in 10 µl distilled water and subjected to qPCR.

Artificial feeding experiments were performed to assess if differences in virus content of both whitefly populations were a result of virus content of the source plants, i.e. higher WmCSV concentrations in watermelon compared to TYLCV in tomato. Purified WmCSV and TYLCV preparations were quantified in a photometer (NanoDrop Spectrophotometer ND-1000) using extinction coefficient of 7.7 published by Goodman and Bird [33] for Bean golden mosaic geminivirus. Each preparation was adjusted to approximately 300 µg purified virus per ml 15% sucrose solution. Three to five days old whiteflies were fed on this solution through Parafilm M (Brand GmbH + Co, Germany) for 48 h under greenhouse conditions (light period 14 h; 24°C/18°C) followed by a transfer to cotton for a two day discharge period. Virus content of the artificial media and individual whiteflies (n = 4 for each virus) were quantified by qPCR. TYLCV measurements were corrected by a factor 2.5 due to normalize for differing virus concentrations in the artificial medium. The experiment was performed with three replicates.

### Quantitative PCR assays and data analysis

Quantitative PCR was carried out using hydrolysis probes (TaqMan) and the Maxima Probe qPCR Master Mix (Fermentas, USA) in a Mastercycler ep *realplex* (Eppendorf, Germany). Primers and probes were designed with Beacon Designer (PREMIER Biosoft International, USA) and supplied by Eurofins MWG Operon GmbH (Germany). Each sample was analyzed in duplicate. For WmCSV, 120 nM of each primer (forward 5′-GTACTTGCAGGCCGTTGAATC-3′; reverse 5′- AAACGGGAGTGGAAATGAGAATATC-3′) and the probe (FAM-5′-CCTGTTCGCTTCGCCATA-3′-TAMRA) were used in a 25 µl reaction volume. For TYLCV, 400 nM of each primer (forward 5′-CGCCCGCCTCGAAGGTTC-3′; reverse 5′- TCGTCGCTTGTTTGTGCCTTG-3′), 1.5 mM of magnesium chloride and 300 nM of the probe (FAM-5′-CGACAGCCCATACAGCAGCCGTG-3′-TAMRA) were used. Cycling parameters were as follows for WmCSV: 50°C for 3 min; 95°C for 5 min; 40 cycles of 95°C for 20 s, 54°C for 30 s, 60°C for 30 s and for TYLCV: 50°C for 3 min; 95°C for 5 min; 40 cycles of 95°C for 15 s, 60°C for 60 s.

For absolute quantification, standard curves were generated from plasmid dilutions carrying WmCSV DNA-A and TYLCV, respectively. Tenfold dilution series were generated and concentration of virus genome molecules was calculated. Standard dilutions of each virus DNA were included in each qPCR run.

To quantify virus in the hemolymph, absolute quantification combined with a relative quantification approach was followed, quantifying 18S rDNA for reference [34]. 560 nM of each primer and the probe were used in a 25 µl reaction volume. The quantification cycle (Cq) for the respective virus gene and 18S rDNA to reach the threshold fluorescence signal level were taken and ΔCq calculated their difference.

Data analysis was performed with the *realplex* software package (Eppendorf, Germany), Microsoft Office Excel (Microsoft Corporation) and SigmaPlot 9.01 (Systat Software GmbH, Germany). The data were plotted on a log scale of 10. Significance of data and standard deviation were calculated with student's *t*-test and *U*-test (Mann-and-Whitney-test).

### Localization of WmCSV and TYLCV with fluorescence *in situ* hybridization

Discharged viruliferous females were aspired after an AAP of five days. Alimentary canals or primary salivary glands were excised on ice, washed in ice cold extraction buffer (136.89 mM sodium chloride, 2.68 mM potassium chloride, 8.09 mM disodium hydrogen phosphate, 1.76 mM potassium dihydrogen phosphate, pH 7.4), fixed in modified Carnoy's fixative ([32], [35]; 60% v/v chloroform, 30% v/v ethanol, 10% v/v glacial acetic acid) for 5 min and washed in hybridization buffer (20 mM Tris-hydrogen chloride, pH 8, 900 mM sodium chloride, 0.01% w/v sodium dodecyl sulfate, 30% v/v formamide). Specimens were hybridized with 10 pM of Cy3-labeled fluorescent oligonucleotide probe (Cy3-5′-GGAACATCAGGGCTTCGATA-3′; Eurofins MWG Operon) in hybridization buffer [28], [36] at 4°C overnight, washed with hybridization buffer and subsequently with double distilled water. The probe designed for the coat protein gene of TYLCV [36] was also suitable for the detection of WmCSV. Nuclei were stained with DAPI (4′,6-diamidino-2-phenylindole). Excised organs of non-viruliferous whiteflies treated similarly and excised organs of viruliferous whiteflies incubated without probe served as controls. At least 20 excised organs were analyzed per treatment.

Specimens were visualized by confocal laser scanning microscopy (Leica TCS SP2, Leica Microsystems Heidelberg GmbH, Germany). Lambda scans were run to confirm the fluorescent signals. Images were collected in z-stacks from 25–35 optical sections of 0.5 µm thickness. Optical sections, maximum intensity projections and overlays were generated using Leica Confocal Software, version 2.5 (Leica Microsystems). Bright light microscopical analyses were performed using differential interference contrast (Axioskop 2 Plus, Carl Zeiss AG, Germany).

### Generation of *a B. tabaci* population incapable to transmit begomoviruses

Individual viruliferous females of *B. tabaci* 95 were transferred to tomato plants for 5 days. Virus infected plants were discarded while plants remaining symptomless and virus free were maintained individually in plexiglas tubes to generate a new population. Offspring were again placed on virus infected plants and subsequently transferred individually to single tomato plants. This procedure was repeated until a population was generated from individual insects that lacked virus transmission. For verification transmission experiments with individual whiteflies (two experiments for each virus) and groups of more than 100 whiteflies (5 experiments for each virus) were performed for TYLCV and for WmCSV. In all experiments *B. tabaci* 95- failed to transmit either of the viruses.

## Results

### Secondary bacterial endosymbionts

*B. tabaci* can harbor several facultative endosymbiotic bacteria. Their impact on *B. tabaci* and role for virus transmission, however, largely remains speculative. To exclude that transmission is affected by abundance of endosymbionts, the endosymbiotic communities of both populations were compared. From six facultative endosymbionts described for *B. tabaci* [32] three were detected in both populations (Table 1). Sequence analysis of amplification products obtained after specific PCR, confirmed their identities as *Rickettsia*, *Hamiltonella* and *Wolbachia*. Most individuals harbored more than one endosymbiont species. *Hamiltonella*, the most abundant facultative endosymbiont in both populations, was detected in 100% males and females of *B. tabaci* 95, 100% males and 80% females of *B. tabaci* 63. *Wolbachia* was abundant in all individuals of *B. tabaci* 95 and present to 95% *in B. tabaci* 63 females and 65% of males. Colonization rates of *Rickettsia* were higher in female individuals (90%) than in male insects (*B. tabaci* 63: 57%, *B. tabaci* 95: 43%). *Arsenophonus*, *Cardinium* and *Fritschea* were not detected.

**Table 1:** Abundance of secondary bacterial endosymbionts in the efficiently transmitting *Bemisia tabaci* 63 and the poor transmitter *B. tabaci* 95.

	*Rickettsia* sp.		*Wolbachia* sp.		*Arsenophonu*s sp.		*Hamiltonella* sp.		*Cardinium* sp.		*Fritschea* sp.	
	*(Rickettsiales)*		*(Rickettsiales)*		*(Enterobacteriales)*		*(Enterobacteriales)*		*(Bacteroidales)*		*(Chlamydiales)*	
	♀	♂	♀	♂	♀	♂	♀	♂	♀	♂	♀	♂
*B. tabaci* 63	90	57	90	65	0	0	80	100	0	0	0	0
*B. tabaci* 95	90	43	100	100	0	0	100	100	0	0	0	0

Adult male and female individuals were examined for the presence of bacterial endosymbionts by PCR (n = 20). Data represent the percentage of whitefly individuals harboring the respective endosymbiont.

### Ingestion rates of WmCSV

**Figure 1:**
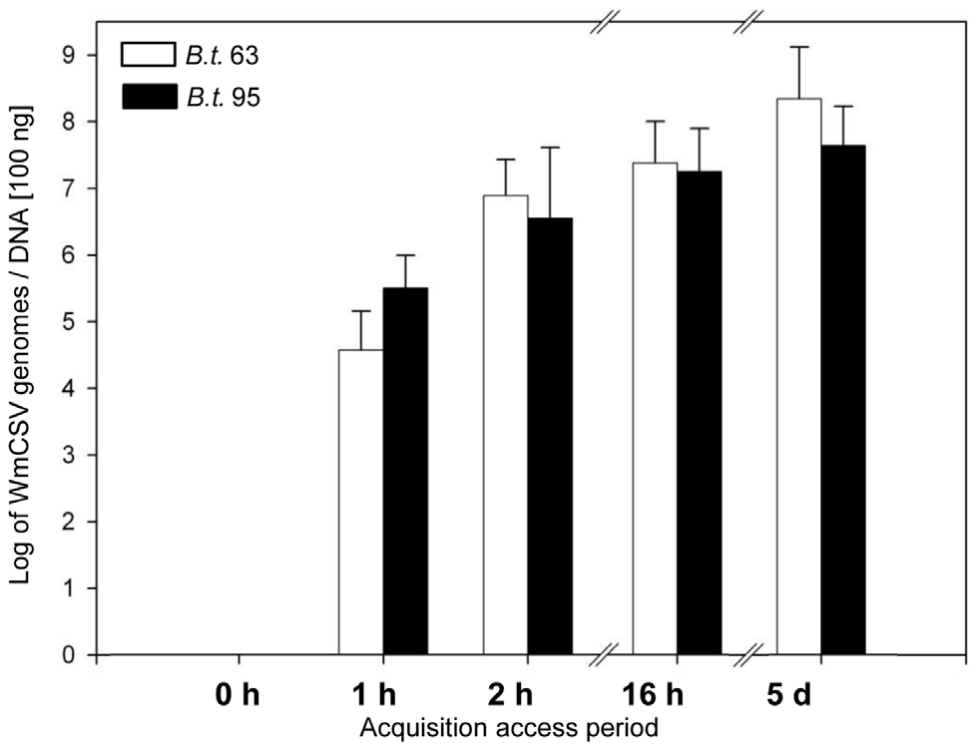
Uptake of WmCSV by the efficient transmitter *Bemisia tabaci* 63 and the poor transmitter *B. tabaci* 95. Virus concentrations were analyzed by qPCR in composite samples of 100 whiteflies of *B. tabaci* 63 (*B.t.* 63) and *B. tabaci* 95 (*B.t.* 95) after a six hour starvation over an acquisition access period of five days (two technical replicates). Median values of virus concentrations were calculated assuming 100 ng DNA as an average DNA content of an individual whitefly. Three independent experiments were performed.

Uptake and accumulation of WmCSV were assessed in insects over 5 days to evaluate whether differences in feeding characteristics of the whitefly populations may have affected virus transmission. Undischarged insects were used for this analysis and thus measurements comprised virus still present in the midgut lumen and virus translocated into the midgut epithelial cells, the hemocoel and the PSGs. After an AAP of one hour 7.6×10^4^/5.1×10^5^ virus genome molecules were found in single individuals of *B. tabaci* 63/95 (Fig. 1). WmCSV concentrations increased during the first 16 h of AAP to 4.7×10^7^/4.2×10^7^ virus genomes per insect (*B. tabaci* 63/95). Whiteflies of both populations acquired comparable virus amounts during this period. After an AAP of 5 days *B. tabaci* 63 accumulated higher amounts of virus (mean value 1.8×10^9^±3.5×10^9^) than the poor transmitter (mean value 1.0×10^8^±1.4×10^8^).

### Quantification of WmCSV and TYLCV in whole insects, excised organs and hemolymph

To follow virus in the insect, virus was quantified by qPCR in discharged whiteflies and calculated for each individual whitefly assuming for reference that an individual whitefly contains approximately 100 ng DNA. This DNA content was found on average from all DNA extractions from groups of whiteflies and calculated for individual insects.

**Figure 2:**
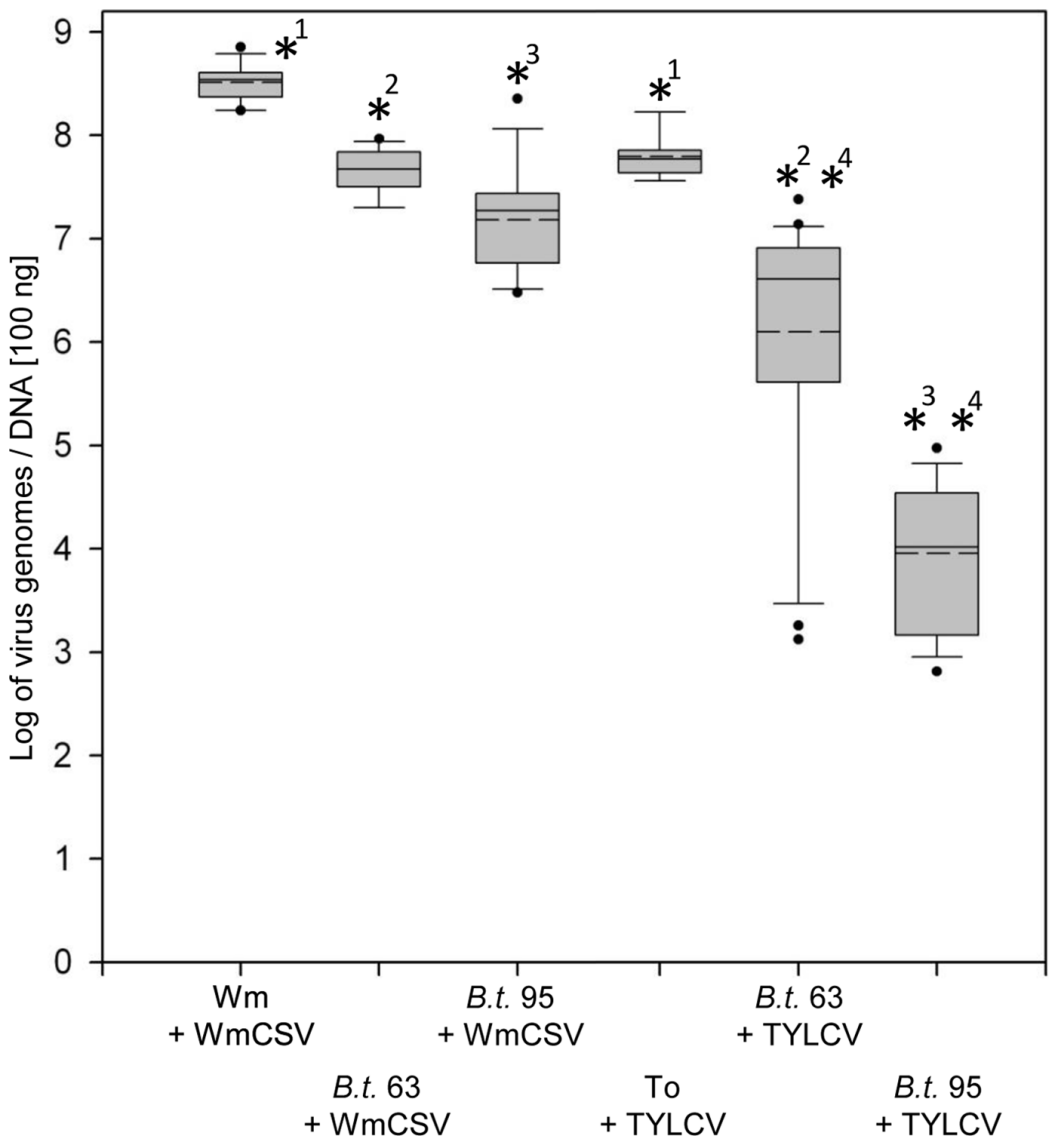
WmCSV and TYLCV concentrations in host plants and insects of *B. tabaci* 63 and 95. Viruses were quantified in watermelon (Wm), tomato (To) and insects (*B.t*.) after an acquisition access period of 5 d by qPCR following 2 d feeding on cotton for discharge of the intestine (n = 18, with two technical replicates each). Virus concentrations were calculated assuming 100 ng DNA as an average DNA content of an individual whitefly. Solid horizontal lines within boxes represent the median; dashed horizontal lines represent mean values; boxes contain values between the 25th and 75th quartiles; the antennae represent 95 percent of all data; dots represent outliers. Asterisks with the same number indicate significant differences between the samples (Student's *t*-test, *p*<0.05).

Concentration of WmCSV in watermelon was significantly higher (6.9×10^6^ genomes per ng DNA; Fig. 2) than TYLCV contents in tomato (5.9×10^5^ genomes per ng DNA, *p*<0.05) and as result a significantly higher uptake of WmCSV compared to TYLCV was measured for both vector populations.

**Figure 3:**
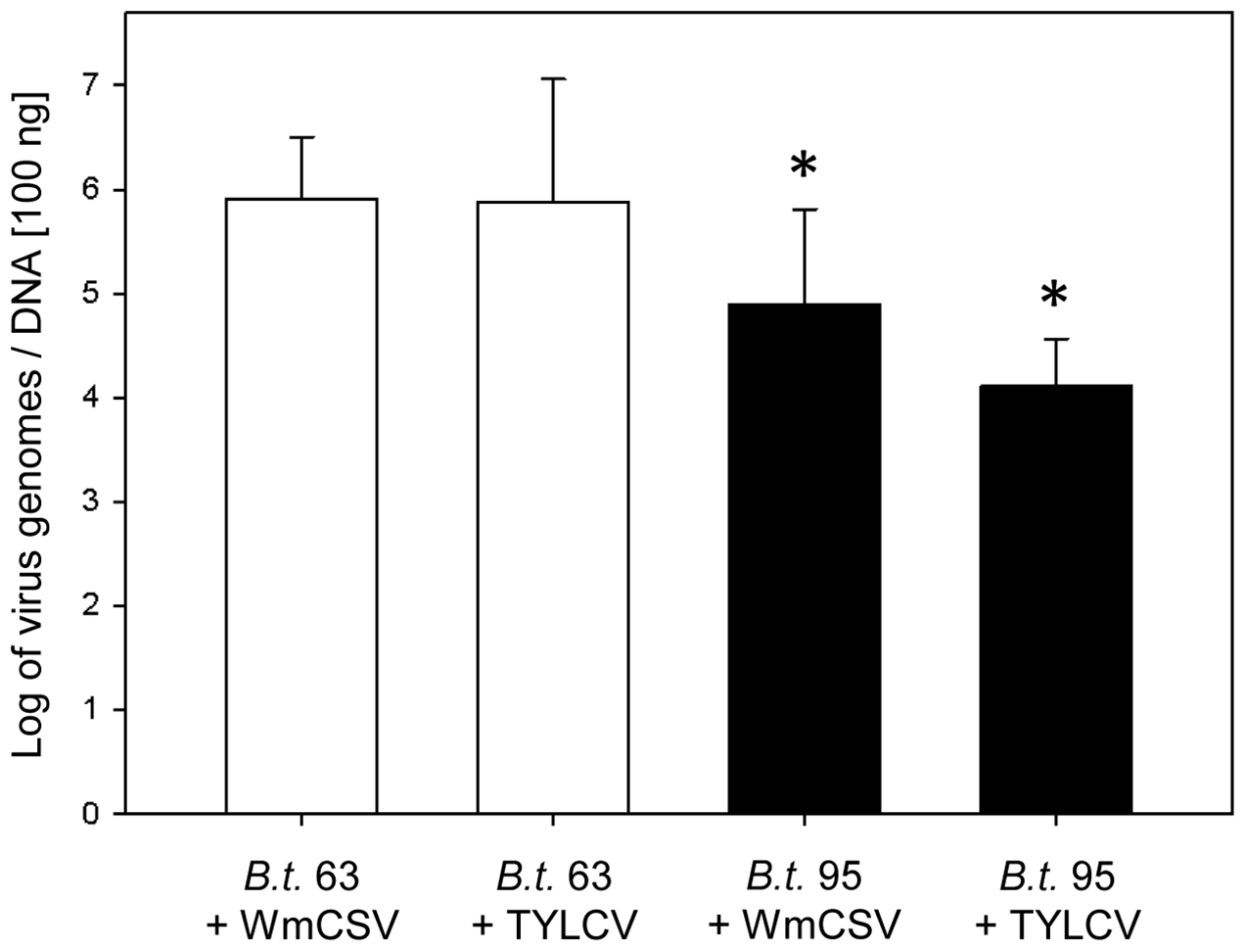
WmCSV and TYLCV concentrations in insects of *B. tabaci* 63 and 95 after artificial feeding. Whiteflies were fed on artificial medium adjusted to similar concentrations of purified WmCSV and TYLCV. Feeding experiments were kept for 48 h under greenhouse conditions followed by two days incubation on cotton for discharge (n = 12, with two technical replicates each). Mean values of virus concentrations were calculated assuming 100 ng DNA as an average DNA content of an individual whitefly. Asterisks indicate significant differences between both whitefly populations having taken up the same virus (Student's *t*-test, *p*<0.05).

Feeding experiments with artificial medium in which concentrations of both viruses were adjusted confirmed that similar amounts of WmCSV and TYLCV were uptaken by *B. tabaci* 63 (Fig. 3). Accordingly, there were no differences in uptake of both viruses by *B. tabaci* 95, however, compared to the efficient transmitter *B tabaci* 63, a significantly (*p*<0.05) lower amount of virus was acquired. The artificial feeding experiments therefore showed that the amount of virus uptake depends on the virus concentration in the source.

**Figure 4:**
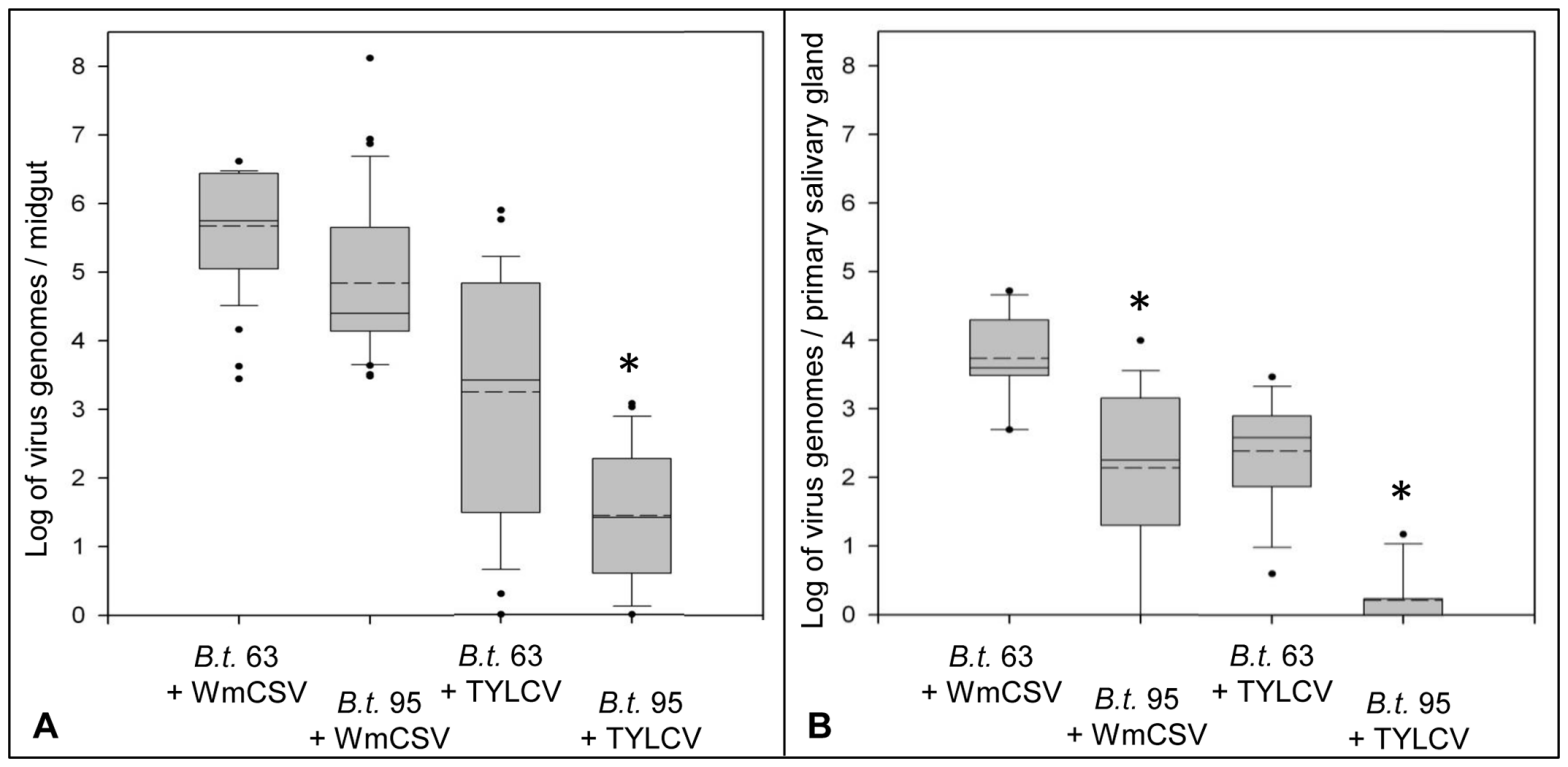
WmCSV and TYLCV concentrations in organs of *B. tabaci* 63 and 95. Viruses were quantified in excised midguts (A) and salivary glands (B) of *B. tabaci* (*B.t*.) after 5 d acquisition access period and 2 d discharge by qPCR (Single midguts represent one sample; n = 36, with two technical replicates each; ten primary salivary glands represent one sample; n = 18, with two technical replicates each). Specification of box plots is given in the legend to Figure 3. Asterisks indicate significant differences between the whitefly populations (Student's *t*-test, *p*<0.05).

Considerably higher concentrations of both viruses were also found in *B. tabaci* 63 (WmCSV/TYLCV: 4.7×10^7^/4.1×10^6^ genomes per whitefly) compared to *B. tabaci* 95 (WmCSV/TYLCV: 1.9×10^7^/1.0×10^4^ virus genomes per whitefly; Fig. 2) after feeding on infected plants, with higher concentrations of WmCSV 4.7×10^7^ virus genomes per *B. tabaci* 63 insect compared to 1.9×10^7^ virus genomes per *B. tabaci* 95 individuum and significant higher amounts of TYLCV with 4.1×10^6^ virus genomes in *B. tabaci* 63 and 1.0×10^4^ virus genomes in *B. tabaci* 95. Accordingly higher amounts of both viruses were detected in excised organs of *B. tabaci* 63 (Fig. 4A). In individual midguts of *B. tabaci* 63 5.6×10^5^/3.6×10^3^ genomes of WmCSV/TYLCV were found compared to 2.6×10^4^/2,6×10^1^ in *B. tabaci* 95. Amounts of virus in the PSGs were significantly lower (*p<*0.05) compared to the respective midguts with 3.9×10^3^/3.8×10^2^ WmCSV/TYLCV genomes in *B. tabaci* 63 and 1.8×10^2^ genomes of WmCSV in *B. tabaci* 95 (Fig. 4B). TYLCV was nearly not detectable in PSG of *B. tabaci* 95 (two virions per PSG in average). In all samples (10 PSG each) WmCSV and TYLCV were detected in *B. tabaci* 63 while only 40% of the samples of *B. tabaci* 95 tested positive for WmCSV. WmCSV and TYLCV were detectable in the hemolymph of *B. tabaci* 63 already after two hours AAP with virus concentrations increasing with feeding time. In *B. tabaci* 95 virus translocation was delayed to 4 h AAP for TYLCV and 6 h AAP for WmCSV. Concentrations of both viruses were significantly higher in *B. tabaci* 63 compared to *B. tabaci* 95 (Table 2). Because it is not possible to collect the entire hemolymph of an insect, the absolute amount of DNA cannot be determined. However, assuming that approximately similar volumes were collected for each whitefly, a comparison between the populations can be done. Table 2 presents a comparison of virus content in the hemolymph under the assumption that 10% and 80% of the hemolymph liquid was collected.

**Table 2:** Translocation of WmCSV and TYLCV into the hemolymph of the efficiently transmitting *Bemisia tabaci* 63 and the poor transmitter *B. tabaci* 95.

AAP [h]	WmCSV		TYLCV	
	Reciprocal of ΔCq +/− Standard deviation		Reciprocal of ΔCq +/− Standard deviation	
	*B. tabaci* 63	*B. tabaci* 95	*B. tabaci* 63	*B. tabaci* 95
0	0.000	0.000	0.000	0.0000
2	0.018+/−0.043	0.000 *	0.092+/−0.085	0.0000 *
4	0.024+/−0.038	0.000 *	0.152+/−0.128	0.056+/−0.050 *
6	0.069+/−0.045	0.012+/−0.030 *	0.111+/−0.086	0.068+/−0.059 *
8	0.072+/−0.012	0.015+/−0.030 *	0.171+/−0.057	0.086+/−0.023 *
24	0.050+/−0.071	0.017+/−0.035 *	0.144+/−0.035	0.022+/−0.044 *
30	0.098+/−0.038	0.040+/−0.041 *	0.167+/−0.027	0.067+/−0.058 *
144	0.121+/−0.029	0.082+/−0.046 *	0.147+/−0.051	0.130+/−0.057 *
	**Estimated virus concentration [virus genomes per whitefly] if 10/80% of the hemolymph was extracted**		**Estimated virus concentration [virus genomes per whitefly] if 10/80% of the hemolymph was extracted**	
144	60000/7500	900/112	600/75	70/9

Hemolymph was collected 0, 2, 4, 6, 8, 24, 30 h and 6 d after transfer of starved whiteflies to infected plants. Virus concentrations in pooled hemolymph of 5 females was analyzed by qPCR in a relative approach with 18S rDNA as reference gene (n = 3, with two technical replicates). ΔCq data of the relative quantification are displayed reciprocally for better illustration. Standard curves of both viruses were included to allow an estimation of absolute virus concentration under the assumption that 10 and 80% of hemolymph was extracted, respectively. Asterisks indicate significant differences between the whitefly populations for one virus at a given time point; Student's *t*-test, *p*<0.05).

### Localization of WmCSV and TYLCV in midguts and primary salivary glands

**Figure 5:**
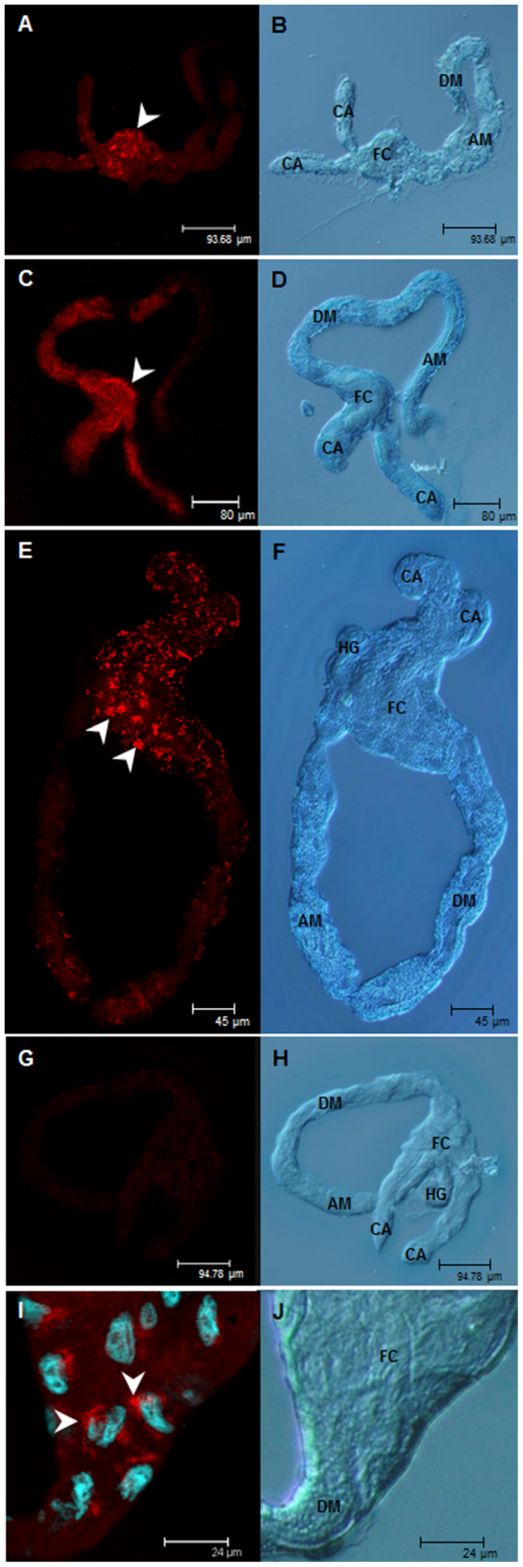
Localization of WmCSV and TYLCV in excised midguts of *B. tabaci* 63 and 95. Viruses were localized after a 5 d acquisition access period and 2 d discharge using fluorescent *in situ* hybridization. Left column: virus localization using specific probes (red); right column: transmitted light images. WmCSV in *B. tabaci* 63 (A, B) and in *B. tabaci* 95 (C, D); TYLCV in *B. tabaci* 63 (E, F) and *B. tabaci* 95 (G, H); WmCSV in *B. tabaci* 63 after fluorescent *in situ* hybridization and nuclei staining with DAPI (I, J). CA, caeca; FC, filter chamber; DM, descending midgut; AM, ascending midgut; HG, hindgut. Arrowheads: virus accumulations.

Fluorescent *in situ* hybridization used to study localization patterns in midgut and PSG traced both viruses predominantly in the filter chamber, caeca and descending midgut (Fig. 5). Both viruses showed a similar distribution pattern with signals in *B. tabaci* 63 generally higher than in *B. tabaci* 95. TYLCV was not detected in midguts of *B. tabaci* 95 due to its low concentrations which also confirmed qPCR results. In general, viruses were found aggregated in clusters within midgut cells and were never found in nuclei, although occasionally surrounding them (Fig. 5I). There was no virus accumulation in terms of a layer in the gut lumen detectable. Results from *in situ* hybridization experiments were confirmed by immunolocalization studies using virus specific antibodies (unpublished data).

**Figure 6:**
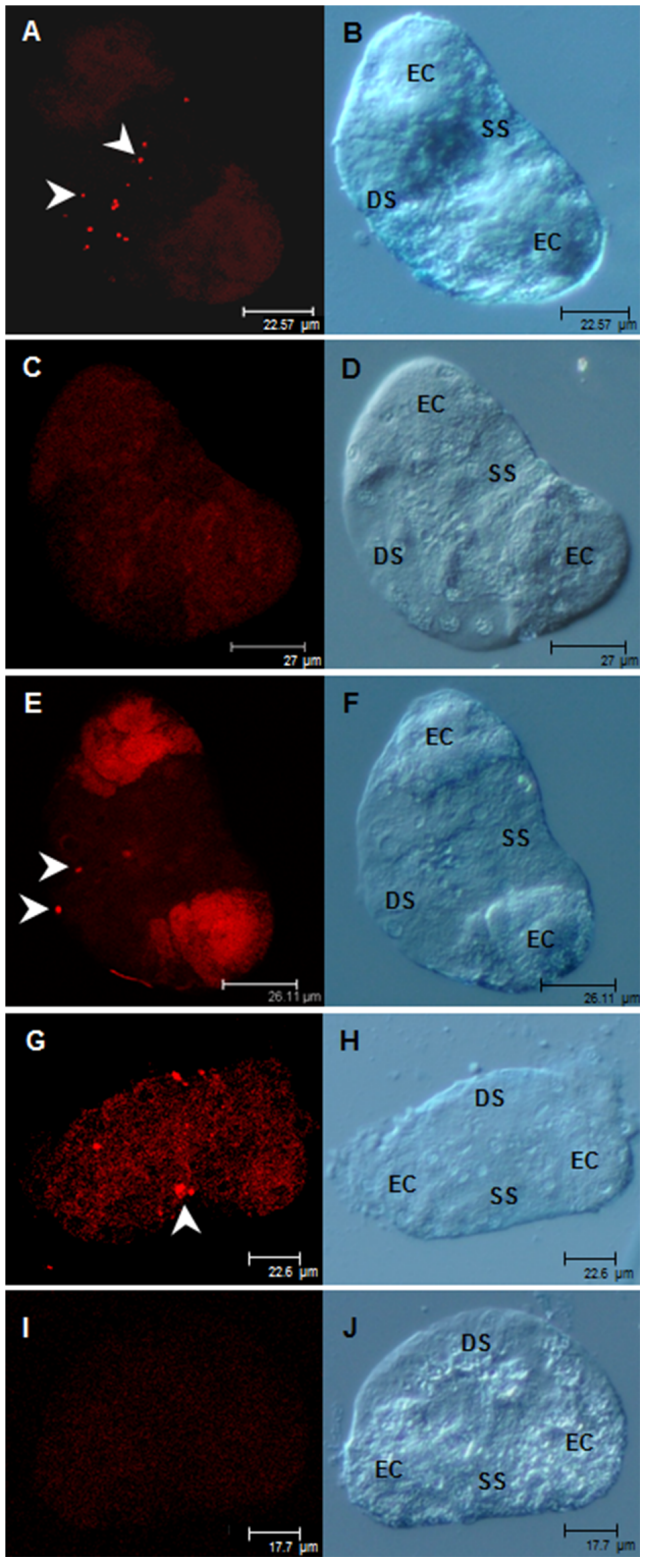
Localization of WmCSV and TYLCV in excised primary salivary glands of *B. tabaci* 63 and 95. Viruses were localized after a 5 d acquisition access period and 2 d discharge by fluorescent *in situ* hybridization. Left column: virus localization using specific probes (red); right column: transmitted light images. WmCSV in *B. tabaci* 63 (A, B) and in *B. tabaci* 95 (C–F); TYLCV in *B. tabaci* 63 (G, H) and *B. tabaci* 95 (I, J). DS, ducal section of the central region; EC, endcap; SS, secretory section of the central region.

In the PSG, fluorescent signals were detected predominantly in the central region (Fig. 6) with similar distribution patterns for both viruses, however, stronger signals for WmCSV due to higher concentrations of this virus. Consistent with the midgut findings, fluorescent signals for TYLCV were not found in PSGs of *B. tabaci* 95. Few PSGs of both *B. tabaci* populations displayed elevated autofluorescence at the endcap region (Fig. 6E) which was easily differentiated from virus signals by lambda scanning. No signals were observed in any control.

### Generation and characterization of a *B. tabaci* 95- population with lack of virus transmission

**Figure 7:**
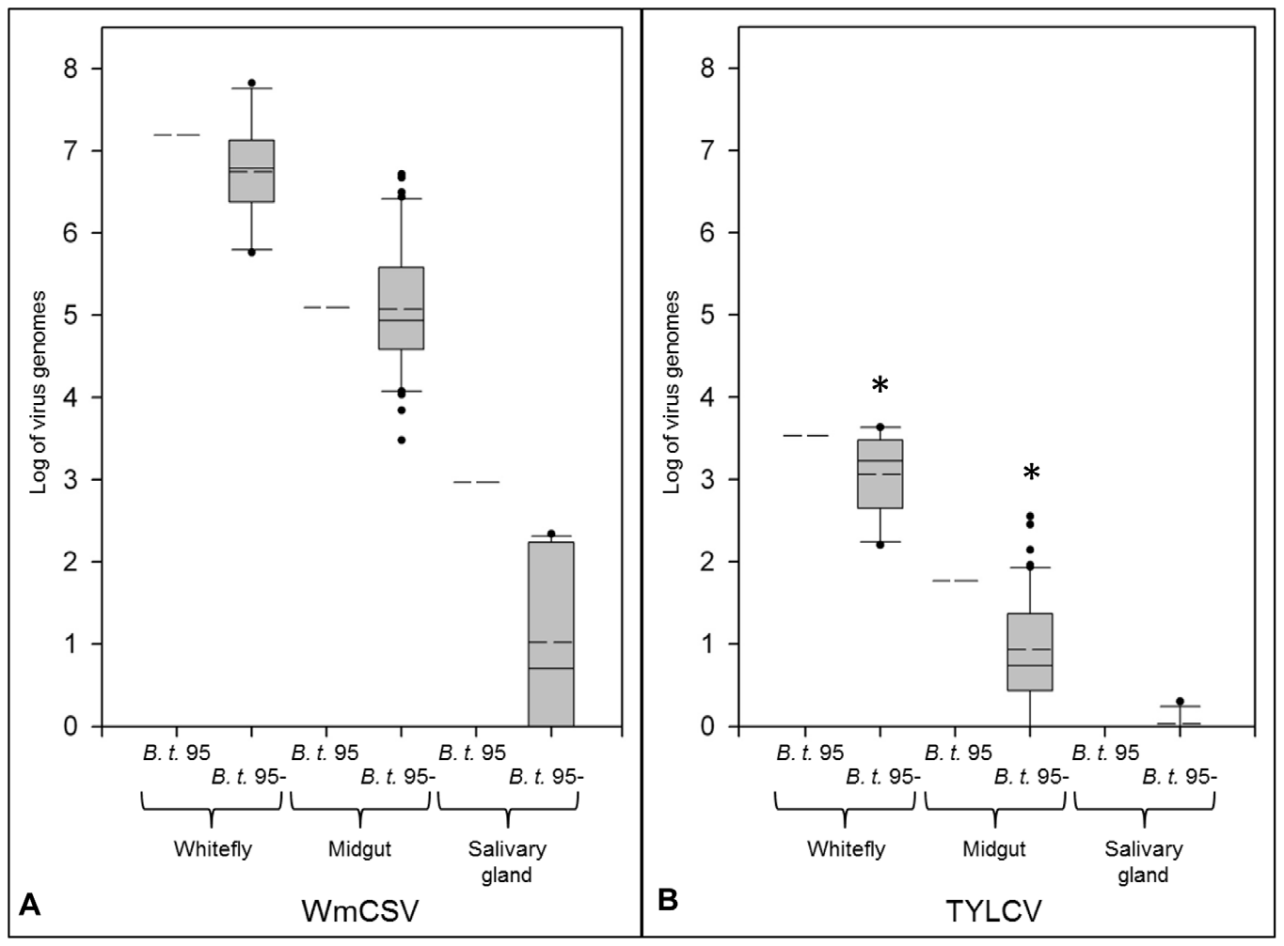
WmCSV and TYLCV concentrations in single whiteflies and excised organs of the poorly transmitting *B. tabaci* 95 and the non-transmitter *B. tabaci* 95-. WmCSV (A) and TYLCV (B) were quantified in individual whiteflies, individual midguts and primary salivary glands (10 primary salivary glands represent one biological sample) after 5 d acquisition access period and 2 d discharge by qPCR (*B. tabaci* 95: n = 4, with two technical replicates each; *B. tabaci* 95-: n = 18 individual whiteflies, n = 36 individual midguts, n = 20 primary salivary glands, with two technical replicates each). Specification of box plots is given in the legend to Figure 2. Asterisks indicate significant differences between the parental population *B. tabaci* 95 and *B. tabaci* 95- (Student's *t*-test, *p*<0.05).

Results from virus quantification and localization analysis of PSGs of *B. tabaci* 95 suggested that the poorly transmitting population comprised a mix of individuals transmitting viruses and others that lacked this feature. Hence virus transmission efficiency appeared to be a consequence of a heterogeneous whitefly population rather than an intrinsic feature of this particular population. Consequently, a population referred to as *B. tabaci* 95- lacking virus transmission was generated following individual female insects and selecting non-transmitting females. The lack of virus transmission was proven in seven independent transmission experiments for each virus. The resulting *B. tabaci* 95- had similar colonization of secondary endosymbionts (♀: 90% *Rickettsia* sp., 100% *Wolbachia* sp., 100% *Hamiltonella* sp.; ♂: 67% *Rickettsia* sp., 100% *Wolbachia* sp., 100% *Hamiltonella* sp.) compared to the parental *B. tabaci* 95 and as predicted, a significant lower concentration of TYLCV in individual insects and excised midguts compared to the parental population (Fig. 7). WmCSV contents were lower in individual insects of *B. tabaci* 95- compared to the parental, although not significantly. In about 50% of the PSG samples negligible amounts of WmCSV were found while TYLCV remained undetectable. Distribution of WmCSV in dissected midguts of *B. tabaci* 95- was as already shown for insects of *B. tabaci* 95 while TYLCV was not traceable by *in situ* hybridization most likely because of low concentrations.

## Discussion

We have investigated the translocation of circular transmitted begomoviruses, WmCSV and TYLCV, in populations of *B. tabaci* 63 and 95 that were chosen for their differential capabilities to transmit the viruses. Recently we found an enhanced transcription of *hsp7*0 in *B. tabaci* 63 after virus uptake and increased virus transmission after antiHSP70 antibody feeding [37]. The results of this study suggested a direct or indirect immune response to virus ingestion and consequently reduced transmission rates. *B. tabaci* 95 responded similarly with an increased *hsp70* transcription after WmCSV and TYLCV uptake (unpublished data) and therefore an elevated immune response can be excluded from being responsible for the low transmission rates.

To test for putative effects of endosymbionts on transmission rates, communities of both populations were compared. Secondary bacterial endosymbionts are abundant in many insects and those can provide additional features for the host [38]. Besides the obligatory bacterial endosymbiont *Portiera aleyrodidarum* [39] *Rickettsia*, *Hamiltonella*, *Wolbachia*, *Arsenophonus*, *Cardinium* and *Fritschea* are described for *B. tabaci* [32], [40], [41] which can occur singly or in mixed communities with others [32], [42]–[44]. Recently, additional *Enterobacter*, *Bacillus*, *Paracoccus, Acinetobacter* and an *Orientia-*like organism were found sporadically in whiteflies [45], [46]. Aside from detection and identification of those secondary endosymbionts, there is only little information on their additional biological functions provided to *B. tabaci.* Several traits are discussed, e.g. the involvement of *Rickettsia* in resistance against various biotic and abiotic stresses [47]–[50], an increase of fitness provided by *Rickettsia*, *Hamiltonella* and *Wolbachia* [51]–[53] and protection against parasitization reported for *Wolbachia* [52].

*Rickettsia* are found localized in bacteriocytes [54], [55] or scattered in the hemocoel [56] in a number of *B. tabaci* populations [32]. Recently, an increased TYLCV transmission efficacy was reported for a *Rickettsia* harboring *B. tabaci* population with enhanced virus uptake and retention compared to an isofemale population lacking *Rickettsia* [57].

A positive effect for virus transmission of *B. tabaci* was also found for *Hamiltonella.* The GroEL expressed by this endosymbiont was found to be enhanced after TYLCV uptake [58] and is assumed to bind to virions and protect them from degradation while passaging through the hemolymph [18], [59]. This was earlier postulated for symbionin, a GroEL like protein found in aphids which protects luteoviruses like the *Potato leafroll virus* from degradation [60]. Recent evidence, however, raised doubts on this GroEL/virus interaction *in vivo* [61]. Notwithstanding, almost all individuals of *B. tabaci* 63 and 95 harbored *Hamiltonella* and those high colonization rates were also reported by Chiel et al. [32] who proposed a mutualistic interaction between this symbiont and its host.

*Wolbachia* is known to alter reproduction processes in many arthropods through induction of parthenogenesis, cytoplasmic incompatibility and male killing [62]–[64]. A *Wolbachia*-mediated antiviral protection was described for *Drosophila* [65]. Influences of *Wolbachia* on the replication of *Chikungunya virus* [66], *Dengue virus* and *West nile virus* in mosquitos [67] and on the vector competence of the insects harboring *Wolbachia* [68], [69] were reported. Tsai et al. [63] showed co-localization of *Wolbachia* and *Japanese encephalitis virus* in mosquito salivary glands but there was no interaction found. The presence of *Wolbachia* in *B. tabaci* MEAM1 species albeit at low frequencies was reported by Chu et al. [70]. In contrast, both *B. tabaci* populations in our study showed high colonization rates with *Wolbachia*.

Although effects of secondary endosymbionts on the efficiency of TYLCV transmission by *B. tabaci* were reported [54]–[56], the actual role or process by which this occurs remains highly speculative. For the two *B. tabaci* populations, a comparison of endosymbiont communities revealed similar colonization with most whitefly individuals harboring mixed communities of *Rickettsia*, *Wolbachia* and *Hamiltonella*. Because density gradient gel electrophoresis (DGGE) of 16S rDNA using individual insects also did not reveal further banding patterns indicating for yet further endosymbionts (unpublished data) it cannot be assumed that colonization with specific endosymbionts is contributing to the differences in transmission rates.

Virus uptake was compared for the whitefly populations to exclude that transmission rates were a result of different feeding behavior patterns [71]. Whiteflies of both populations ingested the same virus amounts during the first 16 h of feeding, however, continuing feeding for 5 days, the efficient transmitter *B. tabaci* 63 accumulated slightly higher virus amounts than *B. tabaci* 95. This was in a comparable range with the report for TYLCV-Mld by Ohnishi et al. [72] who found a maximum of 3.97×10^8^ virus genomes in non-discharged individual insects of *B. tabaci* after 9 days AAP.

The concentration of WmCSV in both whitefly populations was significantly higher than that of TYLCV. This was also found by Polston et al. [73] and Zeidan and Czosnek [74] for TYLCV and another cucurbit begomovirus, Squash leaf curl virus (SLCV), and considered as a specific feature of the virus species. Our artificial feeding experiments confirmed differences in virus uptake between *B. tabaci* populations. However, within each of the *B. tabaci* populations, similar amounts of WmCSV and TYLCV were acquired and there were no differences between the virus species. This proved that virus uptake was not because of virus species differences but a result of virus concentration in the respective host plant. This correlation between virus concentrations of the source plants and virus amounts in whiteflies feeding on these plants was also found by Lapidot et al. [75].

Translocation of begomoviruses in vector and non-vector insects has been studied for several viruses [28], [72], [73], [76]–[78]. The outer and inner membranes of the midgut cells are the first barriers to virus passage and this was also found in our study. In *B. tabaci* 63 virus contents in excised midguts were higher than in *B. tabaci* 95. This is most likely a result of higher rates of virus uptake *B. tabaci* 63. However, virus found in the midgut may also be affected by exocytose activity from midgut to the hemolymph thus virus present in the hemolymph could provide a more precise indication for the translocation process. WmCSV and TYLCV were first detectable in the hemolymph of *B. tabaci* 63 after two hours AAP confirming earlier observations for TYLCV [28], [76] and SLCV [77]. In *B. tabaci* 95 TYLCV was detectable after 4 h and WmCSV only after 6 h, pointing to a delayed virus translocation in the poor transmitter. This delay can be explained by generally lower virus concentrations in the midgut of *B. tabaci* 95. However, the results of our translocation experiments with the two *B. tabaci* 95 and 63 populations rather support the notion that virus translocation into the hemolymph is compromised in *B. tabaci* 95. Despite higher virus concentrations of WmCSV in *B. tabaci* 95 compared to TYLCV concentrations in the efficient virus transmitter *B. tabaci* 63, WmCSV translocation was slower in *B. tabaci* 95. Because collecting hemolymph from *B. tabaci* is not comprehensive and liquids from a number of insects have to be pooled for more precise measurement, it was impossible to correlate results of absolute measurements of virus contents in organs or individual insects with relative qPCR values from hemolymph analysis. Thus the lower amounts of virus in the hemolymph of the poor transmitter compared to the efficiently transmitting population have no absolute reference. Notwithstanding, because both whitefly populations harbored the same endosymbionts, it can be assumed that the reduced virus content in the hemolymph of *B. tabaci* 95 is due to an impaired virus translocation and not the result of virus degradation taking place in *B. tabaci* 95 only.

Studies to reveal localization patterns in the midguts detected WmCSV and TYLCV in filter chamber, caeca and the descending midgut of whiteflies. This was also shown for the begomovirus *Tomato mottle virus* [20], TYLCV and *Tomato yellow leaf curl Sardinia virus* (TYLCSV) [36], [79], [80]. Fluorescent signals were stronger in the efficient transmitter *B. tabaci* 63 compared to *B. tabaci* 95 which was due to the higher virus content in these whiteflies. In the midgut cells of *B. tabaci* 63, WmCSV and TYLCV often appeared in aggregates. Medina et al. [79] using immune gold labeling and electron microscopy found TYLCSV within transport vesicles in midgut cells. This was also found earlier for luteovirus translocation in aphids where receptor-mediated transcytosis through the gut of the vector aphids was postulated and virions enclosed in vesicles were demonstrated [81], [82]. In our studies, vesicles containing virions were never found and hence begomovirus passage through the midgut still remains to be clarified. There was considerably less virus within midgut cells of the weak transmitter *B. tabaci* 95 but virus accumulation in the gut lumen, as reported for TYLCV in the non-vector *Trialeurodes vaporariorum* [72], was not found. Instead, WmCSV and TYLCV accumulated around the nuclei but never in the nuclei of *B. tabaci*. At the PSGs only very little virus was detected, confirming the findings of Czosnek et al. [83] and Rosell et al. [77] that only a small fraction of virus acquired finally succeeds to enter this organ to complete the circulative transmission pathway. In *B. tabaci* 95 virus concentrations at the PSGs were significantly lower compared to *B. tabaci* 63 because of the lower virus concentrations already found in the hemolymph of the poor transmitter. Because we failed to detect virus in groups of PSGs of *B. tabaci* 95 we postulate that a lack or modification of receptors is preventing virus attachment and subsequent translocation into the salivary duct.

Viruses were generally found in the central lumen of the PSG which is in line with immunoelectron microscopical studies of Caciagli et al. [84] demonstrating TYLCSV in the vacuolated area of the PSGs.

From all evidence in this and earlier studies, it is clear that the most substantial amount of virus is withhold in the midgut and as shown in our study TYLCV was almost undetected in the PSGs of *B. tabaci* 95. To dissect the role of the PSGs it is tempting to use the absolute mean values calculated for the virus genomes at the PSGs. Thus on average 629 genome molecules of TYLCV were found in PSGs of *B. tabaci* 63 (virus transmission rates of 80%). While TYLCV was at the detection threshold in the poorly transmitting population *B. tabaci* 95 (virus transmission rates 10%), there were still 1188 genome molecules of WmCSV found at the PSGs of *B. tabaci* 95 insects. It remains still unclear how many virus molecules are required for a successful plant infection, however, the critical role of the PSGs in virus transmission is evident.

The results of these studies suggest that several whitefly genes determine virus uptake and translocation. While genes located in midgut are regulating the rate of virus uptake and gene products represent partial barriers, those expressed in the PSGs seem to be responsible for the final complete barriers to virus transmission. Several genes that were not genetically linked and additively acting were also found in the transmission of *Cereal yellow dwarf virus* by its vector *Shizaphis graminum* [23]. However, while the accessory salivary glands of these aphids only represented incomplete barriers for virus movement the hindgut was identified as the critical and absolute barrier for virus translocation.

Finally, all data of the PSGs of the poorly transmitting population *B. tabaci* 95 indicated that it was a heterogeneous population composed of individuals that were competent and others that were lacking virus transmission capabilities. To prove this, a population *B. tabaci* 95- was generated of which all insects are incompetent of virus transmission. Preliminary experiments revealed only minor variation in endosymbiont colonization compared to the parental population, and reduced virus content in individual insects. This population is now subject to further detailed molecular analysis.

In conclusion, we report the quantification and localization of WmCSV and TYLCV in an efficiently transmitting and a poorly transmitting *B. tabaci* population. Virus uptake was independent of virus species but correlated with virus concentrations of the source plants and whitefly population. Virus translocation from the midgut into the hemolymph was considerably delayed and reduced in the poorly transmitting population and no TYLCV or only insignificant WmCSV were bound to the primary salivary glands. This gives reason to conclude that lack or modification of receptors prevents virus attachment to PSGs or subsequent translocation. We first report the generation of a *B. tabaci* population that lacks whitefly transmission capacity which will allow further studies on the genome features required for circulative transmission of begomoviruses. Meanwhile a *B. tabaci* population 95+ was generated which shows transmission rates for TYLCV between 70–90% (data not shown). This allows direct comparison of an efficiently and a non-transmitting population with the identical genetic background with respect to their virus transmission traits.
